# Identification of Driving *ALK* Fusion Genes and Genomic Landscape of Medullary Thyroid Cancer

**DOI:** 10.1371/journal.pgen.1005467

**Published:** 2015-08-21

**Authors:** Jun Ho Ji, Young Lyun Oh, Mineui Hong, Jae Won Yun, Hyun-Woo Lee, DeokGeun Kim, Yongick Ji, Duk-Hwan Kim, Woong-Yang Park, Hyun-Tae Shin, Kyoung-Mee Kim, Myung-Ju Ahn, Keunchil Park, Jong-Mu Sun

**Affiliations:** 1 Division of Hematology and Oncology, Department of Medicine, Samsung Changwon Hospital, Sungkyunkwan University School of Medicine, Changwon, Korea; 2 Department of Pathology and Translational Genomics, Samsung Medical Center, Sungkyunkwan University School of Medicine, Seoul, Korea; 3 Samsung Genome Institute, Samsung Medical Center, Seoul, Korea; Department of Molecular Cell Biology, Sungkyunkwan University School of Medicine, Suwon, Korea; 4 Molecular Translational Research Center, Samsung Biomedical Research Institute, Seoul, Korea; 5 Division of Hematology and Oncology, Department of Medicine, Samsung Medical Center, Sungkyunkwan University School of Medicine, Seoul, Korea; University of Cincinnati College of Medicine, UNITED STATES

## Abstract

The genetic landscape of medullary thyroid cancer (MTC) is not yet fully understood, although some oncogenic mutations have been identified. To explore genetic profiles of MTCs, formalin-fixed, paraffin-embedded tumor tissues from MTC patients were assayed on the Ion AmpliSeq Cancer Panel v2. Eighty-four sporadic MTC samples and 36 paired normal thyroid tissues were successfully sequenced. We discovered 101 hotspot mutations in 18 genes in the 84 MTC tissue samples. The most common mutation was in the ret proto-oncogene, which occurred in 47 cases followed by mutations in genes encoding Harvey rat sarcoma viral oncogene homolog (N = 14), serine/threonine kinase 11 (N = 11), v-kit Hardy-Zuckerman 4 feline sarcoma viral oncogene homolog (N = 6), mutL homolog 1 (N = 4), Kiesten rat sarcoma viral oncogene homolog (N = 3) and MET proto-oncogene (N = 3). We also evaluated anaplastic lymphoma kinase (*ALK*) rearrangement by immunohistochemistry and break-apart fluorescence *in situ* hybridization (FISH). Two of 98 screened cases were positive for *ALK* FISH. To identify the genomic breakpoint and 5’ fusion partner of *ALK*, customized targeted cancer panel sequencing was performed using DNA from tumor samples of the two patients. Glutamine:fructose-6-phosphate transaminase 1 (*GFPT1)-ALK* and echinoderm microtubule-associated protein-like 4 (*EML4*)*-ALK* fusions were identified. Additional PCR analysis, followed by Sanger sequencing, confirmed the *GFPT1*-*ALK* fusion, indicating that the fusion is a result of intra-chromosomal translocation or deletion. Notably, a metastatic MTC case harboring the *EML4-ALK* fusion showed a dramatic response to an ALK inhibitor, crizotinib. In conclusion, we found several genetic mutations in MTC and are the first to identify *ALK* fusions in MTC. Our results suggest that the *EML4-ALK* fusion in MTC may be a potential driver mutation and a valid target of ALK inhibitors. Furthermore, the *GFPT1-ALK* fusion may be a potential candidate for molecular target therapy.

## Author Summary

Little is known about the molecular biology of medullary thyroid cancer (MTC), which is a rare disease. Genomics are increasingly being used to improve our knowledge about disease biology and to identify therapeutic targets in many cancers. Here, we report the largest genomic results of MTC to date. MTC tissue frequently included several mutations. For the first time, anaplastic lymphoma kinase (*ALK*) rearrangements were detected in MTC: one case with a glutamine:fructose-6-phosphate transaminase 1 (*GFPT1*)*-ALK* fusion, and another case with an echinoderm microtubule-associated protein-like 4 (*EML4*)*-ALK* fusion. The fusion mechanism of the novel *GFPT1-ALK* fusion was successfully investigated using molecular biology techniques. In addition, an inhibitor of ALK (crizotinib) dramatically decreased the number of metastatic MTC lesions harboring the *EML4-ALK* fusion, thus verifying the fusion as a promising target in MTC. Our findings suggest that using rapidly improving sequencing techniques and accumulated genomic data to comprehensively perform genetic analyses on rare tumors, such as MTC, will help to improve the poor prognosis of orphan diseases.

## Introduction

Many cancer gene profiling studies have recently been published, describing genetic trends that are not limited to specific cancers. Next-generation sequencing (NGS) is an important tool for detecting genetic alterations in many kinds of cancers, as it allows for millions of nucleic acid sequences to be simultaneously sequenced within a short period of time and is more cost-effective than older methods. Thus, many researchers and physicians anticipate that NGS will bring the concept of personalized cancer therapy to fruition.

Medullary thyroid cancer (MTC) is a rare malignancy that accounts for up to 3–5% of thyroid cancers. It is derived from calcitonin-secreting para-follicular C cells and can arise in a familial (25%) or sporadic (75%) pattern. Genetic and epigenetic alterations play important roles in the progression and prognosis of MTC [1–3]. Genes encoding the ret proto-oncogene (*RET*) and Ras (*RAS*) are commonly mutated in MTC [4, 5]. The *RET* mutation is believed to be a causative event in both familial and sporadic MTC [6, 7]. In the Mitogen-activated protein kinase (MAPK) pathway, the *RAS* mutation is another genetic rearrangement that is prevalent in sporadic MTC and other types of thyroid cancer [2] but the prevalence and significance of other genetic mutations including *BRAF* in MTC remain unclear.

MTC has a different response to treatment than that of well-differentiated thyroid cancers. Because radioactive iodine does not accumulate in MTC, few therapeutic options are available for advanced MTC. Inhibitors of *RET*, such as cabozantinib and vandetanib, have recently been shown to be effective in advanced MTC [8, 9]. However, whether the *RET* mutation is a predictive factor for the success of these drugs is unclear [9].

Recently, the rearrangement of anaplastic lymphoma kinase (*ALK*) was detected in a small but significant proportion of patients with non-small cell lung cancer (NSCLC) [10]. Several ALK inhibitors, including crizotinib, have achieved dramatic responses in cases of NSCLC harboring *ALK* rearrangements [11–13]. Although *ALK* rearrangement has also been episodically observed in a small set of other cancer types, little is known about *ALK* rearrangements in MTC [14, 15].

In this study, we used targeted NGS and various methods to examine the genetic profiles of MTC and detect *ALK* rearrangements.

## Results

### Basal characteristics and prevalence of gene mutations that are detected by AmpliSeq

**Fig 1:**
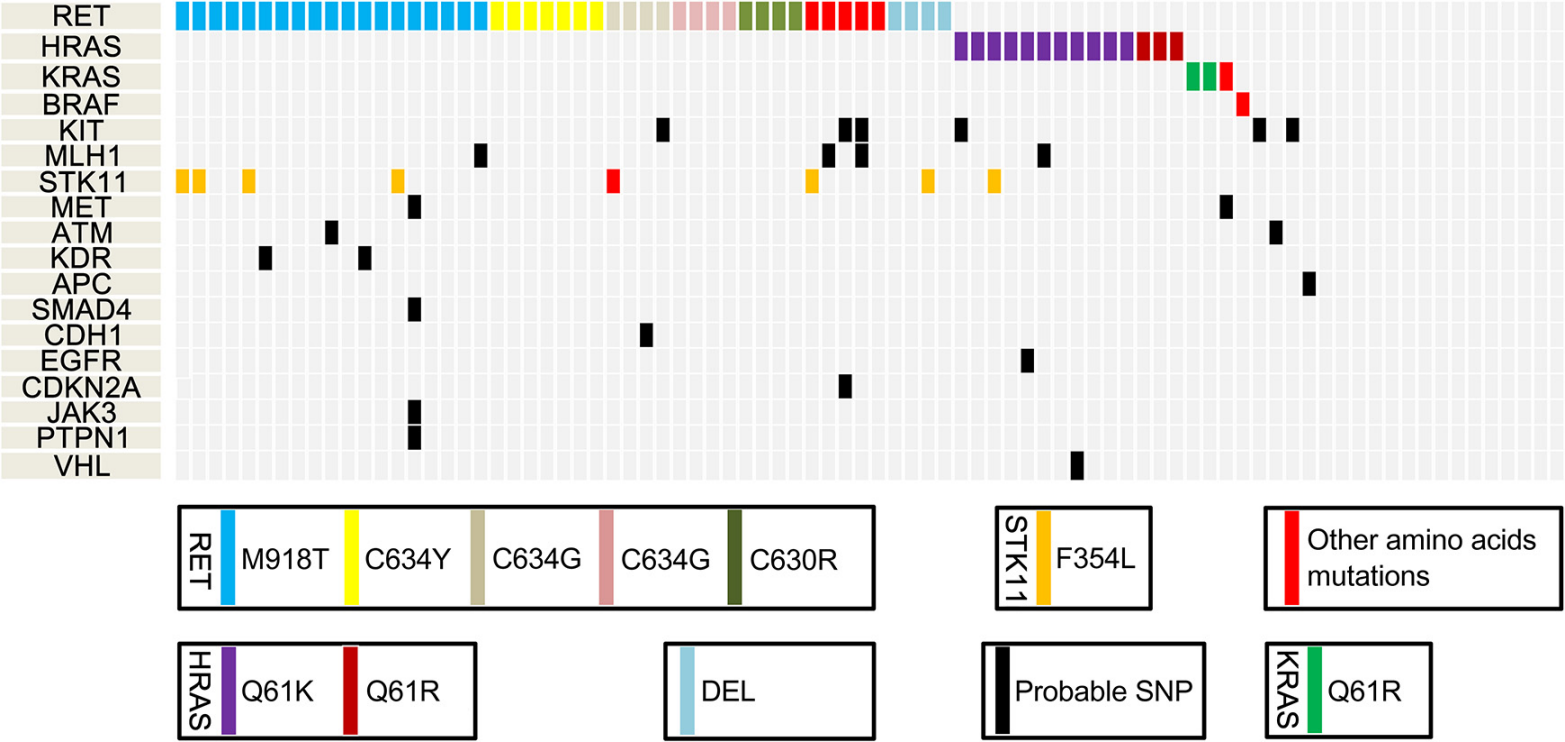
Mutational profiles of medullary thyroid cancer (MTC), as identified by next-generation sequencing. SNP, single-nucleotide polymorphism; DEL, deletion.

Eighty-four samples (11 hereditary, 41 sporadic and 32 unknown) from patients with MTC (mean age of 48.5 years) and 36 paired normal thyroid tissue samples were successfully sequenced. The normal thyroid tissue samples in the MTC patients were used as matched control samples. Of the cases, 32 were male and 52 were female. Detailed demographic, clinic-pathological and genetic characteristics are listed in Table 1 and S2 Table. Hereditary MTCs were defined as cases having either positive germ-line *RET* mutations in blood tests or possession of a strong family history with MTC in at least four family members [16]. The unknown group was composed of MTC cases with no blood *RET* test and no family history of MTC/MEN. The mean value of variant coverage was 593 reads, and the variant coverage ranged from 19 to 1,482 reads. Overall, 101 mutations were observed in the MTC samples. Most mutations (N = 96, 95.0%) were single-nucleotide variants; 5 were deletions. The most common mutation occurred in *RET*, which was observed in 47 cases, followed by mutations in genes encoding Harvey rat sarcoma viral oncogene homolog (N = 14), serine/threonine kinase 11 (N = 11), v-kit Hardy-Zuckerman 4 feline sarcoma viral oncogene homolog (N = 6), mutL homolog 1 (N = 4), Kiesten rat sarcoma viral oncogene homolog (N = 3), MET proto-oncogene (N = 3), ATM serine/threonine kinase (N = 2), kinase insert domain receptor (N = 2), adenomatous polyposis coli (*APC;* N = 2), B-raf proto-oncogene (N = 1), cadherin 1 (N = 1), epidermal growth factor receptor (N = 1), cyclin-dependent kinase inhibitor 2A (*CDKN2A*, N = 1), Janus kinase 3 (N = 1), protein tyrosine phosphatase, non-receptor type 1 (N = 1), SMAD family member 4 (N = 1) and von Hippel-Lundau tumor suppressor (N = 1). We did not detect any dominant gene mutations in 20 MTC samples, which all exhibited wild-type *RET*, *HRAS* and *KRAS*. These are listed in S1 Table and shown in Fig 1.

**Table 1:** Demographic, pathologic and genetic characteristics of medullary thyroid cancer patients.

	Amino acid change in RET		Stage (AJCC 7^th^)		Age (min-max)/ Gender
**Hereditary MTC (11)**	C634Y	3	I	4	35.3 (20–57)
	C634W	4	II	0	M:F 5:6
	D631Y	2	III	5	
	C634G	2	IV	2	
	11 (100%)		N/A	0	
**Sporadic MTC (41)**	M918T	15	I	9	50.3 (22–74)
	Deletion	4	II	4	M:F 19:22
	C630R	2	III	6	
	C634R	1	IV	21	
	C634Y	1	N/A	1	
	C618S	1			
	A883V	1			
	25 (61.0%)				
**Unknown (32)**	M918T	4	I	18	50.6 (17–76)
	C634Y	3	II	3	M:F 8:24
	C630R	2	III	4	
	C634G	2	IV	5	
	11 (34.3%)		N/A	2	

### Specific types of gene mutations

The commonly observed *RET* mutations occurred in exons 10, 11, 15, and 16. Previous studies have shown that M918T is the most common *RET* mutation in MTC [2, 10]. Similarly, M918T (N = 19) was the most common *RET* mutation in our samples, followed by C634Y (N = 7), C634W (N = 4), C634G (N = 4), C630R (N = 4), D631Y (N = 2), and others (N = 7). All *HRAS* mutations occurred in exon 3. The mutant amino acid sequence in each of the *HRAS* mutant cases was Q61K (N = 13). *KRAS* mutations were observed in three cases (Q61R, 2 and G48R, 1), and *BRAF* mutation was found in only one case. The dominant amino acid sequence in *STK11* was F354L (N = 7). Other mutated genes are shown in S1 Table.

### Comparative analysis between MTC tissue and matched normal thyroid tissue

**Fig 2:**
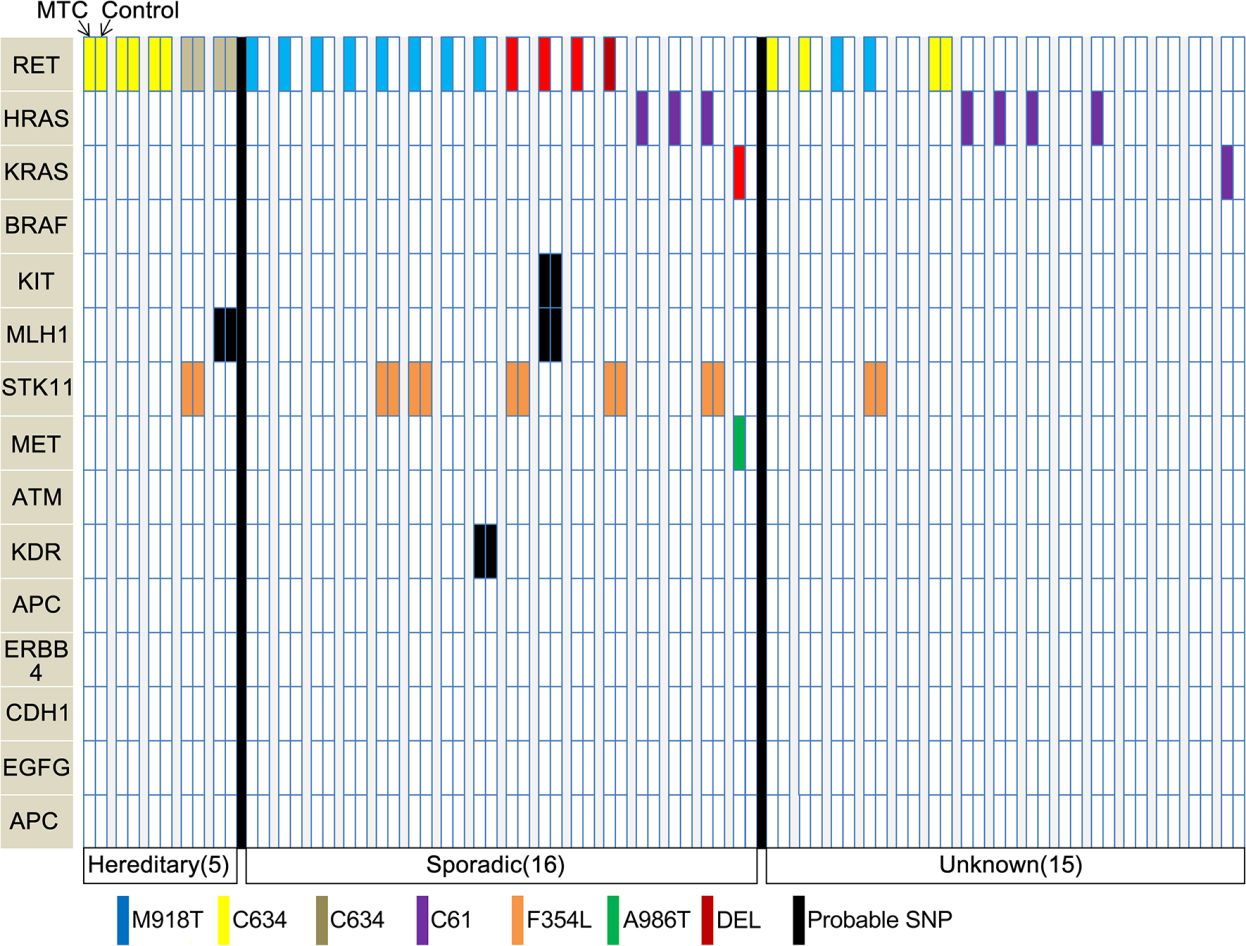
Mutational profiles for comparisons between MTC tissues and normal thyroid tissues from the same individual. Samples from 36 individuals were used. SNP, single-nucleotide polymorphism; DEL, deletion.

We compared the genetic landscapes between 36 MTC tissues and their matched normal thyroid tissues: this group was composed of 16 sporadic, 5 hereditary and 15 cases with unknown information about heredity (Fig 2). In the hereditary MTC cases, *RET* mutations were observed in MTC and their matched normal thyroid tissues: these *RET* mutation types included C634Y, D631Y, and C634W, which are well known to be associated with the MEN2A [17, 18]. One case, which had been classified as an unknown subgroup based on blood test or family history, was found to have C634W mutation in both MTC and normal tissue, leading us to suspect that this case might be hereditary MTC. In 16 sporadic MTC group, several *RET* mutation types (M918T, C630R, C618S and deletion) were detected in MTC tissues, but not in the matched normal thyroid tissues. The M918T *RET* mutations and Q61K *HRAS* mutations were observed only in the MTCs of the sporadic or unknown subgroups, suggesting that these mutations are pathognomonic somatic mutation in MTC. In addition, two *KRAS* (G48R, Q61R) and one *MET* (A986T) mutations were also observed only in MTC tissues. However, *STK11* (F354L), *MLH1*, *KIT*, and *KDR* mutations were observed in both MTC and normal thyroid tissues, which leads their pathognomonic natures unresolved in MTC.

### Screening for *ALK* rearrangement and identification of *ALK* fusion

**Fig 3:**
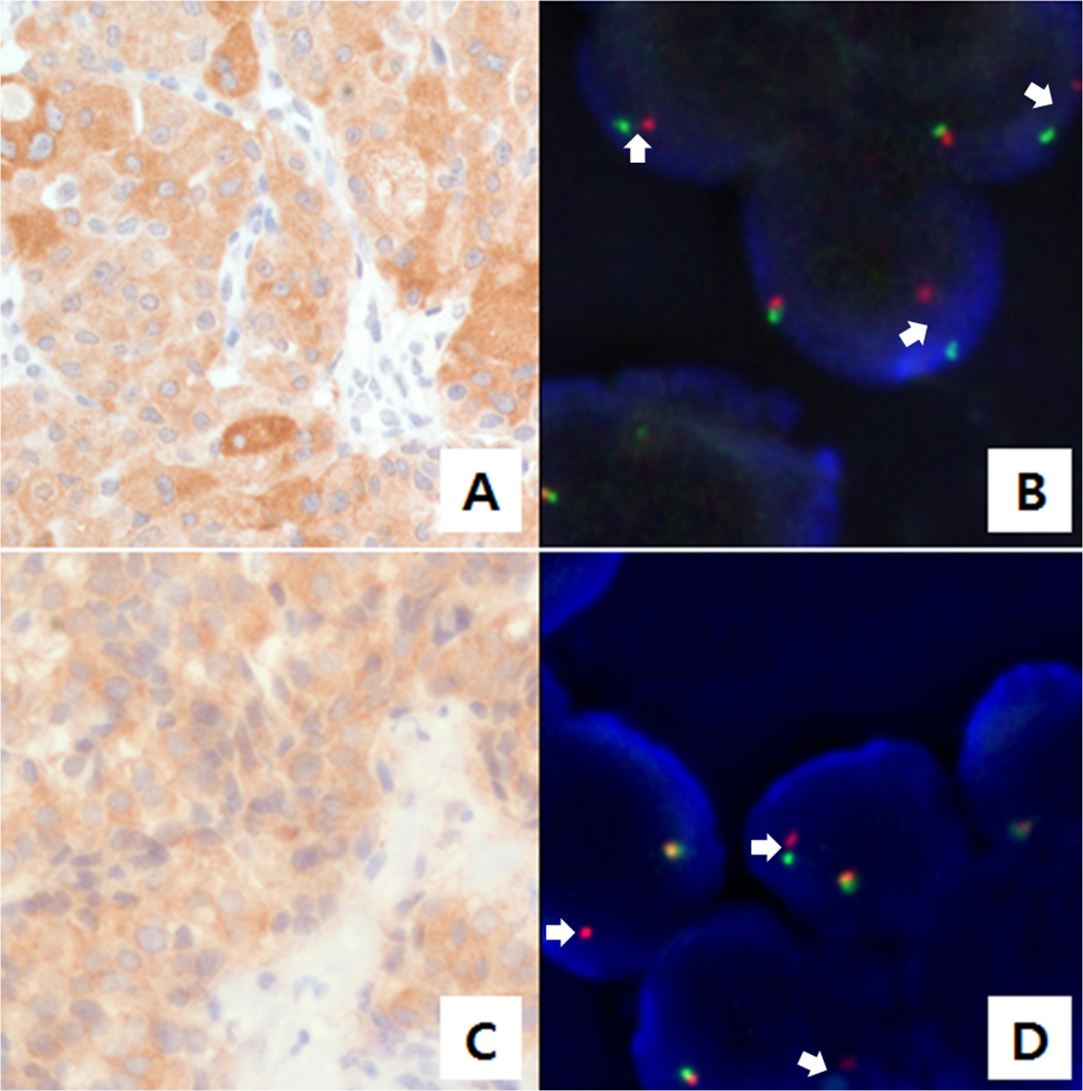
Anaplastic lymphoma kinase (ALK) staining using immunohistochemistry (IHC) and fluorescence *in situ* hybridization (FISH). (A) and (C) ALK staining in tumor cells was detected by IHC. (B) and (D) Results of FISH with the break-apart *ALK* probe are shown. A single red signal or splitting of the red and green signals was observed (marked with arrows). The cases shown in A/B and C/D exhibited glutamine:fructose-6-phosphate transaminase 1 (*GFPT1*)*-ALK* and echinoderm microtubule-associated protein-like 4 (*EML4*)*-ALK* fusions, respectively.

In parallel with targeted sequencing using AmpliSeq, we screened for *ALK* rearrangements. Ninety-eight cases were screened using immunohistochemistry (IHC), and 83 of these cases were also evaluated using AmpliSeq. Nine *ALK*-positive cases were found with IHC scores of 1+ (N = 7), 2+ (N = 1), and 3+ (N = 1). We also performed *ALK* fluorescence *in situ* hybridization (FISH) testing on *ALK*-positive samples that were identified via IHC. The two samples with 2+ and 3+ IHC scores exhibited *ALK* break-apart rearrangements (Fig 3).

**Fig 4:**
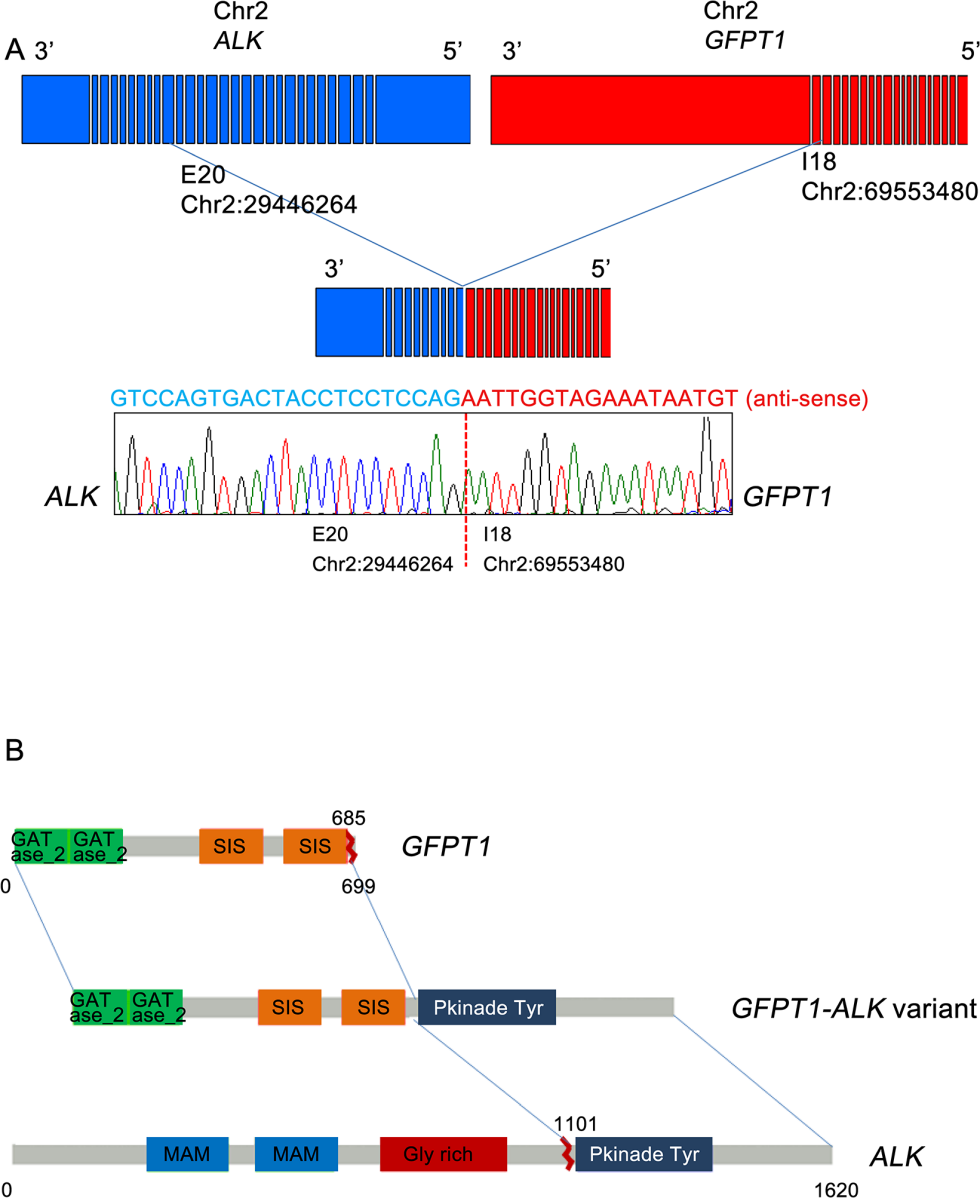
Gene fusion between *GFPT1* and *ALK*. (A) Schematic representation of the fusion of the 5’ *GFPT1* to 3’ *ALK*. (B) The fusion of *GFPT1* to *ALK* is oriented in the same direction and is located in 2p23. Confirmation of the *GFPT1-ALK* fusion was carried out by reverse transcription-polymerase chain reaction and Sanger sequencing. GATase_2, glutamine aminotransferases class-II; SIS, Sugar ISomerase domain; MAM, meprin/A5-protein/PTPmu domain; Gly_rich, glycine rich protein; Pkinase_Tyr, protein tyrosine kinase.

For the two cases harboring *ALK* break-apart rearrangements, targeted cancer panel sequencing (HiSeq 2500, Illumina, USA) was performed to detect the breakpoints and 5’ fusion partner genes of *ALK*. This process revealed two distinct *ALK* fusions. For the first case, a novel fusion gene was detected: 5’ glutamine:fructose-6-phosphate transaminase 1 (*GFPT1*; located in 2p13) was fused to 3’ *ALK* (located in 2p23) with preservation of the *ALK* kinase domain (Fig 4A and 4B). The breakpoints in *GFPT1* and *ALK* were in intron 18 and exon 20, respectively. Based on the gene direction and location, the structural variation was presumed to be intra-chromosomal translocation or deletion. To confirm the fusion, we amplified the genomic fusion point between *GFPT1* and *ALK* using genomic DNA of the MTC tissue. PCR analysis and Sanger sequencing revealed the same results as that of the customized targeted cancer panel (Figs 4B and S1). For the second case, the echinoderm microtubule-associated protein-like 4 (*EML4*)-*ALK* fusion was detected. The breakpoints were located in intron 13 of *EML4* and intron 19 of *ALK*, which indicates that this fusion is the most common variant (E13; A20) in NSCLC [19, 20]. This case exhibited metastatic lesions after thyroidectomy and was enrolled in a Phase I crizotinib trial (NCT01121588). After crizotinib therapy, the tumor lesions in the lung, liver, and bone shrank remarkably, and plasma calcitonin levels decreased. The final results will be disclosed with the full clinical study.

## Discussion

We identified two types of *ALK* fusion genes in MTC by sequencing via IHC, FISH, and NGS analyses. Of the two fusion types, the *EML4-ALK* fusion was the same as the most commonly detected variant in NSCLC, [19] where the *EML4-ALK* fusion is a strong predictive factor for the efficacy of ALK inhibitors [13, 21, 22]. In the current study, the patient with metastatic MTC harboring the *EML4-ALK* fusion showed a dramatic response to crizotinib. We are the first to report an MTC case with a targetable *EML4-ALK* fusion gene. Previously, Kelly et al. used the Illumina HiSeq sequencing system to identify one papillary thyroid cancer case with an *EML4-ALK* fusion [15]. However, they also tested 22 medullary carcinoma cases and did not find any cases with the *EML4-ALK* fusion, as evaluated by reverse transcription-PCR. Their failure to detect the *ALK* rearrangement in MTC is understandable, given that our prevalence rate of *ALK* fusions in the current study was only 2% (2 out of 98 cases). This suggests that more efficient strategies are needed to detect the *ALK* rearrangement. Results from the current study suggest that IHC-based screening, along with FISH-based confirmation and targeted NGS, may be a cost-effective and reliable method to detect *ALK* rearrangements.

Most importantly, we detected a novel *GFPT1-ALK* fusion that has not been reported in any type of cancer. *GFPT1* is a key enzyme in the biosynthesis of N-acetylglucosamine and is required for critical events in neuromuscular transmission [23]. Until now, several fusion partners of *ALK* have been reported in various cancers [24–28]. Among them, huntingdon-interacting protein (*HIP1*)*-ALK* and RAN-binding protein 2 (*RANBP2*)*-ALK*, which have been reported to exist in NSCLC and inflammatory myofibroblastic tumors, respectively, show clinical responses to crizotinib [25, 26]. In the current study, the MTC case harboring the *GFPT1-ALK* fusion showed strong ALK protein expression and did not exhibit co-existing genetic mutations; both of these factors may support an important role for this fusion gene in the pathogenesis of this MTC case. However, we were unable to validate whether *GFPT1-ALK* was a driving oncogene or a therapeutically targetable gene. Whether *GFPT1-ALK* is also a predictor for ALK inhibitors is unclear.

Currently, vandetanib and cabozantinib are approved for the treatment of MTC by the U.S. Food and Drug Administration. However, the prognosis of patients with metastatic MTC is still poor, due to the inherent resistance to radioiodine therapy and aggressive nature of this disease. Furthermore, the rarity of MTC makes it hard to perform prospective studies to find new agents. Therefore, the comprehensive genetic analysis of MTC can help to identify effective ways to improve its prognosis. Despite the low frequency of *ALK* rearrangements in MTC, our techniques can be used to detect target genes in other rare diseases.

In addition, our sequencing analysis of MTC is the largest to date. Previously, Agrawal et al. published the largest genomic analysis of MTC [5], where they performed whole-exome sequencing of 17 sporadic MTCs and 40 additional MTCs (hereditary or sporadic) for validation. *RET* was the dominant mutation (43/57) in that study. We used a larger sample size and accurate verification by comparing 36 pairs of MTC with matched normal thyroid tissues that were acquired from the same person.

In the comparison analyses, all five hereditary cases were observed to have germ-line *RET* mutations in both MTC and control tissues. However, M918T *RET* (N = 10), Q61K *HRAS* (N = 7), *KRAS* (N = 2), and *MET* (N = 1) mutations were harbored dominantly in MTCs. Simbolo et al. identified *RET*, *HRAS*, *KRAS* and *STK11* mutations as significant somatic mutations in MTCs, whereas *TP53*, *KDR*, *KIT*, *MET*, *PIK3CA* and *ATM* mutations were classified as nonpathogenic germ-line variants [29]. Our current data are compatible with that report. Interestingly, however, the F354L *STK11* mutation, regarded as significant somatic mutation by Simbolo et al., was observed in both MTCs and control tissues of our seven cases. Therefore, we presume that the F354L *STK11* mutation is a germ-line mutation in MTC.

In conclusion, we report that the *EML4-ALK* fusion, which was found for the first time in MTC, could be an effective molecular target of crizotinib. Furthermore, our results also suggest that the novel *GFPT1*-*ALK* fusion can be a potential candidate for molecular target therapy. This study included the largest set of molecular profile data in MTC to date, which was achieved by using high-depth NGS panel sequencing, and also presented the genetic landscape of MTC. Further translational research is needed to determine the oncogenic roles of these mutations in MTC.

## Materials and Methods

### Ethics statement

Written informed consent was obtained from all participants, and this study was approved by the Institutional Review Board of Samsung Medical Center. (SMC 2013-02-010).

### Searching for genetic mutation profiles by Ampliseq

We collected data on patients who were histologically diagnosed with MTC without the coexistence of tumors on the parathyroid and adrenal gland. All patients received surgical treatment at Samsung Medical Center between June 2000 and January 2013. Among 101 MTC specimens, 17 were excluded based on quality control (N = 5), preparation failure (N = 11), and sequencing failure (N = 1). The remaining 84 MTC samples were sequenced using an Ion Torrent Personal Genome Machine (IT-PGM, Life Technologies, Grand Island, NY, USA), which takes real-time measurements of hydrogen ions that are produced during DNA replication and allows for rapid sequencing. Eight normal thyroid tissues were obtained by thyroidectomy and sequenced. Mutation profiles between MTC and normal thyroid tissues from eight individuals were compared.

We constructed libraries using the Ion AmpliSeq Panels, Ion AmpliSeq Library Kit, and Ion Xpress Barcodes, as well as 10 ng of DNA sample per pool (Life Technologies). The amplicons were ligated to Ion Adapters and purified. For barcoded library preparations, barcoded adapters from the Ion Xpress Barcode Adapters 1–96 Kit were substituted for the non-barcoded adapter mix in the Ion AmpliSeq Library Kit. Next, the multiplexed barcoded libraries were enriched by clonal amplification using emulsion polymerase chain reaction (PCR) on Ion Sphere Particles (Ion PGM Template 200 Kit) and loaded on an Ion 316 Chip. Massively parallel sequencing was carried out on an Ion PGM using the Ion PGM Sequencing 200 Kit v2. The Ion AmpliSeq Cancer Hotspot Panel v2 covered hotspot regions of 50 oncogenes and tumor suppressor genes.

The primary filtering process was performed with the Torrent Suite v4.0.0 and Ion Torrent Variant Caller v4.0 software and included signal processing, base calling, assigning quality scores, adapter trimming, PCR duplicate removal, read alignment (to human genome reference 19), mapping quality control, coverage analyzing, and variant calling [30]. To detect variants, a minimum coverage of 100 reads was achieved with a cutoff value of at least 5% in the variant calling rate (frequency). Variant calls were further analyzed by using ANNOVAR variant filtering and COSMIC database (dbSNP build 137) annotating, and these analyses were based on changes in the amino acid sequence.

### *ALK* immunohistochemistry (IHC) and fluorescence *in situ* hybridization (FISH)

The *ALK* IHC assay used a mouse monoclonal *ALK* antibody (5A4, Novocastra, Newcastle, United Kingdom) and the antibody for *ALK* was diluted to 1:30, treated, and incubated at 42°C for 2 hours. *ALK* IHC scores were assigned as follows: 0, no staining; 1+, faint or weak staining intensity with more than 5% tumor cells or any staining intensity with ≤5% tumor cells; 2+, moderate cytoplasmic reactivity with more than 5% tumor cells; and 3+, granular cytoplasmic reactivity of strong intensity in more than 5% of tumor cells [31]. Cases that showed *ALK*-positive staining with a score of 1+ or greater were analyzed by FISH with the Vysis *ALK* Break-Apart FISH Probe Kit (Abbott Laboratories, Abbott Park, IL). Samples were considered positive for *ALK* FISH if more than 15% of cells were positive or an isolated red signal (IRS) in tumor cells.

### Customized targeted cancer panel sequencing for *ALK* fusion genes

Genomic DNA extraction was performed using the QIAamp DNA mini kit (Qiagen, Valencia, CA, USA), according to the manufacturer’s instructions. The Nanodrop 8000 UV-Vis spectrometer (Thermo Scientific Inc., DE, USA), Qubit 2.0 Fluorometer (Life Technologies), and 2200 TapeStation Instrument (Agilent Technologies, Santa Clara, CA, USA) were used to check the concentration, purity, and degradation of extracted genomic DNA. For the next step, samples that passed our quality control thresholds were used.

Genomic DNA (250 ng) from the tissues was sheared by the Covaris S220 (Covaris, Woburn, MA, USA) and used for the construction of the library using customized RNA baits and the SureSelect XT reagent kit, HSQ (Agilent Technologies), according to the manufacturer’s protocol. The customized RNA baits covered whole exons and flanking intronic sequences of the 83 genes. After enriched exome libraries were multiplexed, the libraries were sequenced on the HiSeq 2500 sequencing platform (Illumina, USA), as described previously [32]. Briefly, a paired-end DNA sequencing library was prepared through the following processes: genomic DNA shearing, end-repair, A-tailing, paired-end adaptor ligation, and amplification. After the library was hybridized with bait sequences for 16 hours, the captured library was purified and amplified with an index barcode tag. Then, the quality and quantity of the captured library were measured. Sequencing of the exome library was carried out using the 100-bp, paired-end mode of the TruSeq Rapid PE Cluster kit and TruSeq Rapid SBS kit (Illumina, San Diego, CA, USA).

### PCR for *ALK* fusion genes

The newly identified glutamine:fructose-6-phosphate transaminase 1 (*GFPT1*)*-ALK* fusion gene was detected by targeted cancer panel sequencing, and its respective genomic rearrangement was confirmed by genomic PCR analysis, followed by Sanger sequencing. Genomic DNA was isolated from formalin-fixed, paraffin-embedded (FFPE) tumor samples using a ReliaPrep FFPE genomic DNA extraction kit (Promega, Madison, WI, USA). The PCR products were indicative of fusion points within intron 18 of *GFPT1* and exon 20 of *ALK*, based on target sequencing results. PCR analysis of genomic DNA for *GFPT1-ALK* was performed with a pair of primers flanking the putative fusion point: *GFPT1* F (5’-TCTGTGTGAACTGGCACCTT-3’) and *ALK* R (5’-ATTCAGCCCCTACACTGCAC-3’). PCR products were then separated on a 2% E-Gel SizeSelect agarose gel (Invitrogen, Carlsbad, CA, USA). For genomic PCR controls, we used DNA from the same FFPE tumor samples with glyceraldehyde-3-phosphate dehydrogenase PCR primers. In reactions that produced a PCR product of the expected size, the amplicons underwent gel purification and sequencing using a 3130 XL ABI Prism sequencer (Applied Biosystems, Foster City, CA, USA) with Bigdye Terminator v3.1 Cycle sequencing kits, according to the manufacturer’s instructions.

## Supporting Information

S1 Fig Nucleotide sequence of the glutamine:fructose-6-phosphate transaminase 1 (*GFPT1*)–anaplastic lymphoma kinase (*ALK*) fusion, as determined by Sanger sequencing. (TIF)

S1 Table Overall Ampliseq results in the entire medullary thyroid cancer (MTC) cohort. (XLS)

S2 Table Clinical, demographic and pathological characteristics of entire medullary thyroid cancer (MTC) cohort. (DOCX)
