# Supplementary figures and images for: Identification of Driving ALK Fusion Genes and Genomic Landscape of Medullary Thyroid Cancer
Source: PLoS Genet. 2015 Aug 21;11(8):e1005467. doi: 10.1371/journal.pgen.1005467 (PMC4546689; doi:10.1371/journal.pgen.1005467)

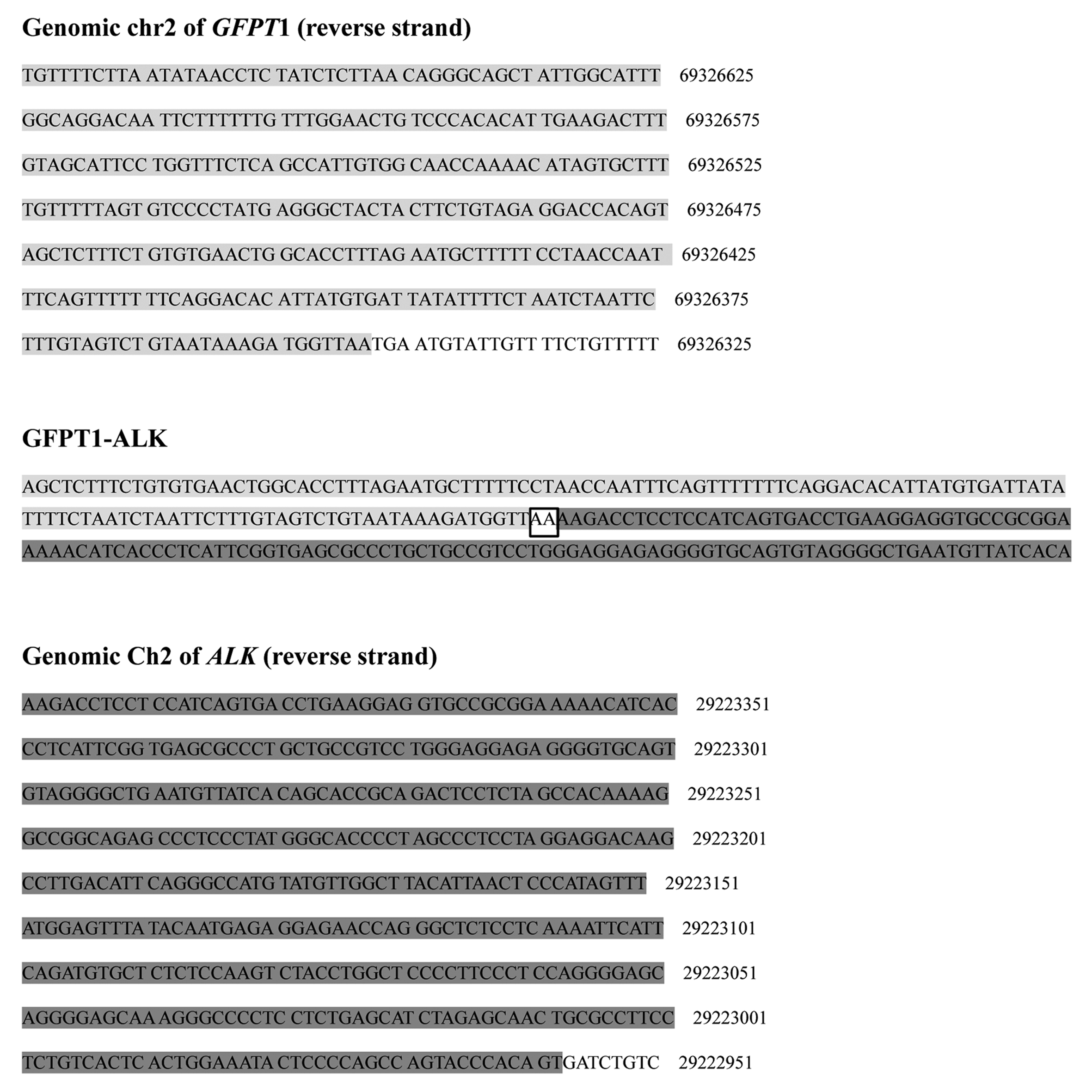

Supplement: S1 Fig — (TIF) [file pgen.1005467.s001.tif]
