# Supplementary material for: Identification of Driving ALK Fusion Genes and Genomic Landscape of Medullary Thyroid Cancer
Source: PLoS Genet. 2015 Aug 21;11(8):e1005467. doi: 10.1371/journal.pgen.1005467 (PMC4546689; doi:10.1371/journal.pgen.1005467)
Supplement: S2 Table — (DOCX) [file pgen.1005467.s003.docx]

| **No** | **Gender** | **Age** | **Calcitonin* (pg/ml)** | **Symptom at Diagnosis** | **TNM stage** | **Heredity** |
| --- | --- | --- | --- | --- | --- | --- |
| 1 | F | 55 | 3830 | - | T4aN0M0 | Sporadic |
| 2 | F | 66 | 373 | Mass | T3N0M0 | Unknown |
| 3 | F | 24 | 8 | Mass | T2N0M0 | Unknown |
| 4 | F | 30 | 70 | Fatigue | T1aN0M0 | Hereditary |
| 5 | M | 28 | 214 | Mass | T2N1bM0 | Hereditary |
| 6 | F | 58 | 1150 | - | T2N0M0 | Unknown |
| 7 | F | 22 | 97500 | Mass | T4aN1bM0 | Sporadic |
| 8 | F | 50 | 66 | - | T1bN0M0 | Sporadic |
| 9 | M | 57 | 750 | Mass | T2N0M0 | Sporadic |
| 10 | F | 73 | 283 | - | T1bN0M0 | Unknown |
| 11 | M | 50 | 44 | - | T1aN0M0 | Unknown |
| 12 | F | 53 | 136 | Headche | T3N0M0 | Hereditary |
| 13 | F | 34 | 3230 | Strong family history | T3N0M0 | Hereditary |
| 14 | F | 53 | 191 | Weight loss | T1bN0M0 | Sporadic |
| 15 | M | 34 | 7360 | Mass | T4aN1bM0 | Sporadic |
| 16 | F | 49 | 1690 | - | T2N0M0 | Sporadic |
| 17 | F | 32 | 33600 | Mass | T4aN1bM0 | Sporadic |
| 18 | F | 53 | 66 | Fatigue | T1aN0M0 | Unknown |
| 19 | F | 43 | 97 | - | T1aN0M0 | Sporadic |
| 20 | F | 58 | 289 | - | T1aN0M0 | Unknown |
| 21 | M | 41 | 251 | Mass | T4aN1bM0 | Sporadic |
| 22 | M | 32 | 880 | Mass | T1bN1aM0 | Hereditary |
| 23 | F | 63 | 17200 | Mass | T4aN1bM0 | Sporadic |
| 24 | F | 68 | 1060 | Mass | T4aN1bM0 | Sporadic |
| 25 | F | 41 | 1970 | Mass | T1bN0M0 | Unknown |
| 26 | F | 44 | 1082 | - | T2N1bM0 | Unknown |
| 27 | F | 68 | 173 | Weight loss | N/A | Unknown |
| 28 | F | 29 | 2210 | Mass | T3N0M0 | Hereditary |
| 29 | F | 54 | 94 | Mass | N/A | Unknown |
| 30 | M | 48 | 2020 | Mass | T3N1bM0 | Sporadic |
| 31 | M | 51 | 3 | Mass | T2N1bM0 | Sporadic |
| 32 | M | 57 | 252 | Mass | T1bN1bM0 | Hereditary |
| 33 | F | 70 | 7200 | - | T1bN1bM0 | Sporadic |
| 34 | M | 33 | 760 | Mass | T3N1bM0 | Sporadic |
| 35 | M | 45 | 820 | - | T2N0M0 | Unknown |
| 36 | F | 58 | 340 | - | T3N1bM0 | Unknown |
| 37 | M | 43 | 115 | Mass | T1aN0M0 | Sporadic |
| 38 | F | 30 | 144 | Mass | T2N1bM0 | Sporadic |
| 39 | F | 46 | 21 | Mass | T1aN0M0 | Unknown |
| 40 | F | 48 | 1 | Mass | T2NxM0 | Sporadic |
| 41 | F | 47 | 2140 | - | T2N1bM0 | Sporadic |
| 42 | F | 53 | 237 | - | T1aN0M0 | Unknown |
| 43 | F | 51 | 6 | - | T1aN0M0 | Unknown |
| 44 | F | 38 | 90 | - | T1aN1aM0 | Sporadic |
| 45 | F | 50 | 48 | Mass | T1bN0M0 | Sporadic |
| 46 | F | 42 | 11 | - | T1aN0M0 | Sporadic |
| 47 | M | 54 | 15 | - | T1aN1aM0 | Unknown |
| 48 | M | 60 | 2359 | Mass | T2N1aM0 | Sporadic |
| 49 | F | 41 | 270 | Mass | T2N0M0 | Sporadic |
| 50 | F | 51 | 1370 | Strong family history | T1bN0M0 | Hereditary |
| 51 | M | 48 | 399 | - | T1bN1bM0 | Sporadic |
| 52 | M | 62 | 256 | - | T1bN1bM0 | Sporadic |
| 53 | M | 76 | 7 | - | T1bN1bM0 | Unknown |
| 54 | F | 58 | 174 | - | T1bN0M0 | Unknown |
| 55 | M | 60 | 9 | - | T1aN0M0 | Unknown |
| 56 | F | 40 | 45000 | - | T1bN0M0 | Unknown |
| 57 | M | 70 | 118 | - | T2N1bM0 | Sporadic |
| 58 | M | 46 | 296 | - | T1bN1bM0 | Sporadic |
| 59 | F | 54 | 45000 | - | T1bN1bM0 | Sporadic |
| 60 | M | 26 | 19 | Strong family history | T1aN0M0 | Hereditary |
| 61 | M | 37 | 7683 | - | T1aN0M0 | Unknown |
| 62 | F | 55 | 42 | - | T1aN0M0 | Sporadic |
| 63 | M | 58 | 325 | - | T1bN1aM0 | Sporadic |
| 64 | M | 40 | 380 | - | T1bN1bM0 | Sporadic |
| 65 | F | 57 | 93 | - | T1aN0M0 | Sporadic |
| 66 | F | 28 | 882 | Strong family history | T1bN0M0 | Hereditary |
| 67 | F | 50 | 2434 | - | T2N1bM0 | Unknown |
| 68 | M | 71 | 631 | - | T2N0M0 | Sporadic |
| 69 | F | 53 | 34 | - | T1aN0M0 | Unknown |
| 70 | M | 45 | 217 | - | T1bN0M0 | Unknown |
| 71 | M | 62 | 125 | - | T3N1bM0 | Sporadic |
| 72 | F | 53 | 920 | - | T1bN1bM0 | Unknown |
| 73 | F | 43 | 16 | - | T1aN0M0 | Unknown |
| 74 | M | 74 | 65 | - | T1aN0M0 | Sporadic |
| 75 | M | 51 | 134 | - | T1aN1aM0 | Sporadic |
| 76 | F | 57 | 141 | - | T1aN0M0 | Unknown |
| 77 | M | 55 | 635 | - | T3N1aM0 | Unknown |
| 78 | M | 20 | 298 | Hypertension | T1aN1aM0 | Hereditary |
| 79 | F | 34 | 502 | Mass | T1bN0M0 | Unknown |
| 80 | F | 46 | 257 | - | T3N1aM0 | Unknown |
| 81 | F | 57 | 5000 | - | T3N1aM0 | Sporadic |
| 82 | F | 17 | N/A | Mass | T1aN0M0 | Unknown |
| 83 | F | 35 | 873 | - | T2N1bM0 | Sporadic |
| 84 | M | 55 | 17450 | mass | T4aN1bM1 | Sporadic |

*Serum calcitonin level at diagnosis of medullary thyroid cancer
